# Pf7: an open dataset of *Plasmodium falciparum* genome variation in 20,000 worldwide samples

**DOI:** 10.12688/wellcomeopenres.18681.1

**Published:** 2023-01-16

**Authors:** MalariaGEN, Muzamil Mahdi Abdel Hamid, Mohamed Hassan Abdelraheem, Desmond Omane Acheampong, Ambroise Ahouidi, Mozam Ali, Jacob Almagro-Garcia, Alfred Amambua-Ngwa, Chanaki Amaratunga, Lucas Amenga-Etego, Ben Andagalu, Tim Anderson, Voahangy Andrianaranjaka, Ifeyinwa Aniebo, Enoch Aninagyei, Felix Ansah, Patrick O Ansah, Tobias Apinjoh, Paulo Arnaldo, Elizabeth Ashley, Sarah Auburn, Gordon A Awandare, Hampate Ba, Vito Baraka, Alyssa Barry, Philip Bejon, Gwladys I Bertin, Maciej F Boni, Steffen Borrmann, Teun Bousema, Marielle Bouyou-Akotet, Oralee Branch, Peter C Bull, Huch Cheah, Keobouphaphone Chindavongsa, Thanat Chookajorn, Kesinee Chotivanich, Antoine Claessens, David J Conway, Vladimir Corredor, Erin Courtier, Alister Craig, Umberto D'Alessandro, Souleymane Dama, Nicholas Day, Brigitte Denis, Mehul Dhorda, Mahamadou Diakite, Abdoulaye Djimde, Christiane Dolecek, Arjen Dondorp, Seydou Doumbia, Chris Drakeley, Eleanor Drury, Patrick Duffy, Diego F Echeverry, Thomas G Egwang, Sonia Maria Mauricio Enosse, Berhanu Erko, Rick M. Fairhurst, Abdul Faiz, Caterina A Fanello, Mark Fleharty, Matthew Forbes, Mark Fukuda, Dionicia Gamboa, Anita Ghansah, Lemu Golassa, Sonia Goncalves, G L Abby Harrison, Sara Anne Healy, Jason A Hendry, Anastasia Hernandez-Koutoucheva, Tran Tinh Hien, Catherine A Hill, Francis Hombhanje, Amanda Hott, Ye Htut, Mazza Hussein, Mallika Imwong, Deus Ishengoma, Scott A Jackson, Chris G Jacob, Julia Jeans, Kimberly J Johnson, Claire Kamaliddin, Edwin Kamau, Jon Keatley, Theerarat Kochakarn, Drissa S Konate, Abibatou Konaté, Aminatou Kone, Dominic P Kwiatkowski, Myat P Kyaw, Dennis Kyle, Mara Lawniczak, Samuel K Lee, Martha Lemnge, Pharath Lim, Chanthap Lon, Kovana M Loua, Celine I Mandara, Jutta Marfurt, Kevin Marsh, Richard James Maude, Mayfong Mayxay, Oumou Maïga-Ascofaré, Olivo Miotto, Toshihiro Mita, Victor Mobegi, Abdelrahim Osman Mohamed, Olugbenga A Mokuolu, Jaqui Montgomery, Collins Misita Morang’a, Ivo Mueller, Kathryn Murie, Paul N Newton, Thang Ngo Duc, Thuy Nguyen, Thuy-Nhien Nguyen, Tuyen Nguyen Thi Kim, Hong Nguyen Van, Harald Noedl, Francois Nosten, Rintis Noviyanti, Vincent Ntui-Njock Ntui, Alexis Nzila, Lynette Isabella Ochola-Oyier, Harold Ocholla, Abraham Oduro, Irene Omedo, Marie A Onyamboko, Jean-Bosco Ouedraogo, Kolapo Oyebola, Wellington Aghoghovwia Oyibo, Richard Pearson, Norbert Peshu, Aung P Phyo, Christopher V Plowe, Ric N Price, Sasithon Pukrittayakamee, Huynh Hong Quang, Milijaona Randrianarivelojosia, Julian C Rayner, Pascal Ringwald, Anna Rosanas-Urgell, Eduard Rovira-Vallbona, Valentin Ruano-Rubio, Lastenia Ruiz, David Saunders, Alex Shayo, Peter Siba, Victoria J Simpson, Mahamadou S. Sissoko, Christen Smith, Xin-zhuan Su, Colin Sutherland, Shannon Takala-Harrison, Arthur Talman, Livingstone Tavul, Ngo Viet Thanh, Vandana Thathy, Aung Myint Thu, Mahamoudou Toure, Antoinette Tshefu, Federica Verra, Joseph Vinetz, Thomas E Wellems, Jason Wendler, Nicholas J White, Georgia Whitton, William Yavo, Rob W van der Pluijm

**Affiliations:** 1 Institute of Endemic Diseases, University of Khartoum, Khartoum, Sudan; 2 Nuclear Applications In Biological Sciences, Sudan Atomic Energy Commission, Khartoum, Sudan; 3 Department of Biomedical Sciences, School of Allied Health Sciences, University of Cape Coast, Cape Coast, Ghana; 4 Health Research Epidemiological Surveillance and Training Institute (IRESSEF), Université Cheikh Anta Diop, Dakar, Senegal; 5 Wellcome Sanger Institute, Hinxton, UK; 6 Medical Research Council Unit The Gambia at the London School of Hygiene and Tropical Medicine, Banjul, The Gambia; 7 National Institute of Allergy and Infectious Diseases (NIAID), NIH, Maryland, USA; 8 West African Centre for Cell Biology of Infectious Pathogens (WACCBIP), University of Ghana, Legon, Ghana; 9 Navrongo Health Research Centre, Ghana Health Service, Navrongo, Ghana; 10 United States Army Medical Research Directorate-Africa, Kenya Medical Research Institute/Walter Reed Project, Kisumu, Kenya; 11 Texas Biomedical Research Institute, San Antonio, USA; 12 Université d'Antananarivo, Antananarivo, Madagascar; 13 Health Strategy and Delivery Foundation, Lagos, Nigeria; 14 Department of Biomedical Sciences, School of Basic and Biomedical Sciences, University of Health & Allied Sciences, Ho, Ghana; 15 University of Buea, Buea, Cameroon; 16 Instituto Nacional de Saúde (INS), Maputo, Mozambique; 17 Mahidol-Oxford Tropical Medicine Research Unit (MORU), Bangkok, Thailand; 18 Centre for Tropical Medicine and Global Health, University of Oxford, Oxford, UK; 19 Menzies School of Health Research, Charles Darwin University, Darwin, Northern Territory, Australia; 20 Nuffield Department of Medicine, University of Oxford, UK; 21 Institut National de Recherche en Santé Publique, Nouakchott, Mauritania; 22 National Institute for Medical Research (NIMR), Dar es Salaam, Tanzania; 23 Department of Epidemiology, International Health Unit, Universiteit Antwerpen, Antwerp, Belgium; 24 Walter and Eliza Hall Institute, Melbourne, Australia; 25 Deakin University, Geelong, Australia; 26 Burnet Institute, Melbourne, Australia; 27 KEMRI Wellcome Trust Research Programme, Kilifi, Kenya; 28 Institute of Research for Development (IRD), Paris, France; 29 Oxford University Clinical Research Unit (OUCRU), Ho Chi Minh City, Vietnam; 30 Institute for Tropical Medicine, University of Tübingen, Tübingen, Germany; 31 London School of Hygiene and Tropical Medicine, London, UK; 32 Radboud University Medical Center, Nijmegen, The Netherlands; 33 Department of Parasitology-Mycology, Université des Sciences de la Santé, Libreville, Gabon; 34 NYU School of Medicine Langone Medical Center, New York, USA; 35 Department of Pathology, University of Cambridge, Cambridge, UK; 36 National Center for Parasitology, Entomology and Malaria Control, Phnom Penh, Cambodia; 37 Center of Malariology, Parasitology and Entomology (CMPE), Vientiane, Lao People's Democratic Republic; 38 Mahidol University, Bangkok, Thailand; 39 LPHI, MIVEGEC, INSERM, CNRS, IRD, University of Montpellier, Montpellier, France; 40 National University of Colombia, Bogota, Colombia; 41 Liverpool School of Tropical Medicine, Liverpool, UK; 42 Malawi-Liverpool-Wellcome Trust Clinical Research Program, Blantyre, Malawi; 43 Malaria Research and Training Centre, University of Science, Techniques and Technologies of Bamako, Bamako, Mali; 44 WorldWide Antimalarial Resistance Network – Asia Regional Centre, Bangkok, Thailand; 45 University Clinical Research Center (UCRC), Bamako, Mali; 46 Departamento de Microbiología, Universidad del Valle, Cali, Colombia; 47 Centro Internacional de Entrenamiento e Investigaciones Médicas - CIDEIM, Cali, Colombia; 48 Biotech Laboratories, Kampala, Uganda; 49 Aklilu Lemma Institute of Pathobiology, Addis Ababa University, Addis Ababa, Ethiopia; 50 National Institutes of Health (NIH), Maryland, USA; 51 Dev Care Foundation, Dhaka, Bangladesh; 52 Broad Institute of Harvard and MIT and Harvard, Cambridge, MA, USA; 53 Department of Immunology and Medicine, US Army Medical Component, Armed Forces Research Institute of Medical Sciences (USAMC-AFRIMS), Bangkok, Thailand; 54 Laboratorio ICEMR-Amazonia, Laboratorios de Investigacion y Desarrollo, Facultad de Ciencias y Filosofia, Universidad Peruana Cayetano Heredia, Lima, Peru; 55 Nogouchi Memorial Institute for Medical Research, Legon-Accra, Ghana; 56 Wellcome Centre for Human Genetics, University of Oxford, Oxford, UK; 57 Department of Entomology, Purdue University, West Lafayette, USA; 58 Centre for Health Research & Diagnostics, Divine Word University, Madang, Papua New Guinea; 59 University of South Florida, Tampa, USA; 60 Department of Medical Research, Yangon, Myanmar; 61 East African Consortium for Clinical Research (EACCR), Dar es Salaam, Tanzania; 62 Center for Applied Genetic Technologies, University of Georgia, Athens, GA, USA; 63 The University of Calgary, Calgary, Canada; 64 U.S. Military HIV Research Program, Walter Reed Army Institute of Research, Silver Spring, MD, USA; 65 University Félix Houphouët-Boigny, Abidjan, Cote d'Ivoire; 66 Myanmar Oxford Clinical Research Unit, University of Oxford, Yangon, Myanmar; 67 University of Public Health, Yangon, Myanmar; 68 University of Georgia, Athens, USA; 69 Medical Care Development International, Maryland, USA; 70 National Institute of Allergy and Infectious Diseases, Phnom Penh, Cambodia; 71 University Gamal Abdel Nasser of Conakry, Conakry, Guinea; 72 Institut National de Santé Publique, Conakry, Guinea; 73 Harvard TH Chan School of Public Health, Harvard University, Boston, USA; 74 Lao-Oxford-Mahosot Hospital-Wellcome Trust Research Unit, Microbiology Laboratory, Mahosot Hospital, Vientiane, Lao People's Democratic Republic; 75 Institute of Research and Education Development (IRED), University of Health Sciences, Ministry of Health, Vientiane, Lao People's Democratic Republic; 76 Bernhard Nocht Institute for Tropical Medicine, Hamburg, Germany; 77 Research in Tropical Medicine, Kwame Nkrumah University of Sciences and Technology, Kumasi, Ghana; 78 MRC Centre for Genomics and Global Health, Big Data Institute, Oxford University, Oxford, UK; 79 Juntendo University, Tokyo, Japan; 80 Department of Biochemistry and Centre for Biotechnology and Bioinformatics, University of Nairobi, Nairobi, Kenya; 81 Faculty of Medicine, University of Khartoum, Khartoum, Sudan; 82 Department of Paediatrics and Child Health, University of Ilorin, Ilorin, Nigeria; 83 World Mosquito Program, Monash University, Melbourne, Australia; 84 University of Melbourne, Melbourne, Australia; 85 National Institute of Malariology, Parasitology and Entomology (NIMPE), Hanoi, Vietnam; 86 MARIB - Malaria Research Initiative Bandarban, Bandarban, Bangladesh; 87 Medical University of Vienna, Vienna, Austria; 88 Shoklo Malaria Research Unit, Mahidol-Oxford Tropical Medicine Research Unit, Faculty of Tropical Medicine, Mahidol University, Mae Sot, Thailand; 89 Eijkman Institute for Molecular Biology, Jakarta, Indonesia; 90 King Fahid University of Petroleum and Minerals (KFUMP), Dhahran, Saudi Arabia; 91 KEMRI Centres for Disease Control and Prevention (CDC) Research Program, Kisumu, Kenya; 92 Centre for Bioinformatics and Biotechnology, University of Nairobi, Nairobi, Kenya; 93 Kinshasa School of Public Health, University of Kinshasa, Kinshasa, Congo, Democratic Republic; 94 Institut de Recherche en Sciences de la Santé, Ouagadougou, Burkina Faso; 95 Nigerian Institute of Medical Research, Lagos, Nigeria; 96 Parasitology and Bioinformatics Unit, Faculty of Science, University of Lagos, Lagos, Nigeria; 97 College of Medicine, University of Lagos, Lagos, Nigeria; 98 Shoklo Malaria Research Unit, Bangkok, Thailand; 99 University of Maryland School of Medicine, Maryland, USA; 100 Institute of Malariology, Parasitology, and Entomology (IMPE) Quy Nhon, Ministry of Health, Quy Nhon, Vietnam; 101 Institut Pasteur de Madagascar, Antananarivo, Madagascar; 102 Universités d'Antananarivo et de Mahajanga, Antananarivo, Madagascar; 103 Cambridge Institute for Medical Research, University of Cambridge, Cambridge, UK; 104 World Health Organization (WHO), Geneva, Switzerland; 105 Institute of Tropical Medicine Antwerp, Antwerp, Belgium; 106 Universidad Nacional de la Amazonia Peruana, Iquitos, Peru; 107 Department of Medicine, Uniformed Services University, Bethesda, MD, USA; 108 Nelson Mandela Institute of Science and Technology, Arusha, Tanzania; 109 Papua New Guinea Institute of Medical Research, Goroka, Papua New Guinea; 110 Center for Vaccine Development and Global Health, University of Maryland, School of Medicine, Baltimore, MD, USA; 111 MIVEGEC, Université de Montpellier, IRD, CNRS, Montpellier, France; 112 Department of Microbiology and Immunology, Columbia University Irving Medical Center, New York, NY, USA; 113 University of Kinshasa, Kinsasha, Congo, Democratic Republic; 114 Sapienza University of Rome, Rome, Italy; 115 Yale School of Medicine, New Haven, CT, USA; 116 Seattle Children’s Hospital, Seattle, USA; 117 Malaria Research and Control Center of the National Institute of Public Health, Abidjan, Cote d'Ivoire

**Keywords:** malaria, plasmodium falciparum, genomics, data resource, genomic epidemiology

## Abstract

We describe the MalariaGEN Pf7 data resource, the seventh release of *Plasmodium falciparum* genome variation data from the MalariaGEN network.  It comprises over 20,000 samples from 82 partner studies in 33 countries, including several malaria endemic regions that were previously underrepresented.  For the first time we include dried blood spot samples that were sequenced after selective whole genome amplification, necessitating new methods to genotype copy number variations.  We identify a large number of newly emerging *crt* mutations in parts of Southeast Asia, and show examples of heterogeneities in patterns of drug resistance within Africa and within the Indian subcontinent.  We describe the profile of variations in the C-terminal of the *csp* gene and relate this to the sequence used in the RTS,S and R21 malaria vaccines.  Pf7 provides high-quality data on genotype calls for 6 million SNPs and short indels, analysis of large deletions that cause failure of rapid diagnostic tests, and systematic characterisation of six major drug resistance loci, all of which can be freely downloaded from the MalariaGEN website.

## Introduction

Despite global malaria eradication efforts in the mid-20th century and more recent advances in malaria control, *Plasmodium falciparum* remains endemic throughout Africa, Asia, South America and Oceania. According to the most recent World Malaria Report, each year over 200 million people suffer from malaria due to *P. falciparum* and over 600,000 die as a result ^1^. Most of the disease burden falls on Africa, and particularly African children. There is international commitment to control malaria more effectively and many countries are working towards the long-term goal of malaria elimination. However the parasites are continually evolving to resist antimalarial drugs and to evade host immunity, and this is a major challenge to sustainable malaria control and elimination.

Our understanding of the evolutionary biology and population genomics of malaria parasites has advanced considerably over the past decade. There is now a substantial body of literature on the genomic diversity and global population structure of malaria parasites, on within-host genetic variation and what this tells us about superinfection and cotransmission, on the identification and monitoring of parasite drug resistance loci, on genetic variation in malaria vaccine antigens, and on methods of analysing genetic relatedness to understand patterns of malaria transmission. A useful summary of the current state of the field can be found in the recent review by Neafsey *et al.* ^2^.

This rapidly growing area of research is underpinned by open data on genome sequence variation in natural parasite populations. Here we report a new release of curated open data on *Plasmodium falciparum* genome variation from the MalariaGEN network ^3^. It includes samples featured in previous MalariaGEN data releases including the Pf3k Project ^4^, the *Plasmodium falciparum* Community Project ^5^ and the GenRe Mekong Project ^6^. To avoid confusion between different MalariaGEN datasets we now identify each by a version number. In this new nomenclature, the previous version ^5^ is called Pf6, and the version described here is called Pf7.

Whole genome sequencing of all the samples in the Pf7 dataset was performed at the Wellcome Sanger Institute and a standardised analysis pipeline was used for variant discovery and genotyping. The Pf7 analysis pipeline was broadly similar to that used for the Pf6 dataset with some improvements that are described in more detail below. Sequence data and genotype calls were returned to partners for use in their own analyses and publications in line with MalariaGEN’s guiding principles on equitable data sharing ^3^.

The Pf7 dataset comprises 20,864 samples of *P. falciparum* collected by 82 partner studies from 33 countries in Africa, Asia, South America and Oceania between 1984 and 2018 ( Table 1, Supplementary Tables 1 and 2, Supplementary Figure 1). Compared to the Pf6 dataset, this includes 13,752 new samples, 33 new partner studies and 5 additional countries. The majority of new samples (12,146) were collected since 2014, but there were also 379 samples collected prior to 2000 (Supplementary Figure 2).

**Table 1.** Counts of samples in the dataset. Countries are grouped into ten major sub-populations based on their geographic and genetic characteristics. For each country, the table reports: the number of distinct sampling locations (first-level administrative divisions); the total number of samples sequenced; the number of high-quality samples included in the analysis; the percentage of samples collected between 2016–2018, the most recent sampling period in the dataset; and the percentage of samples which are new since the Pf6 release. There are 20,704 samples from natural infections with validated metadata and 160 classified as unverified identity, where this information is not available. The breakdown by admin division is reported in Supplementary Table 1 and the list of contributing studies in Supplementary Table 2.

Major sub-population	Country	Sampling locations	Sequenced samples	Analysis set samples	% analysis samples 2016-2018	% analysis samples new in Pf7
**SA (South America)**	**Peru**	1	21	21	0%	0%
	**Colombia**	4	159	135	59%	88%
	**Venezuela**	1	2	2	50%	100%
**AF-W (Africa - West)**	**Gambia**	3	1,247	863	13%	74%
	**Senegal**	2	155	150	0%	44%
	**Guinea**	2	199	151	0%	1%
	**Mauritania**	3	104	92	0%	18%
	**Côte d'Ivoire**	1	71	71	0%	1%
	**Mali**	6	1,804	1,167	38%	63%
	**Burkina Faso**	1	58	57	0%	2%
	**Ghana**	7	4,145	3,131	54%	73%
	**Benin**	2	334	150	76%	76%
	**Nigeria**	2	140	110	74%	74%
	**Gabon**	1	59	55	0%	100%
	**Cameroon**	1	294	264	11%	11%
**AF-C (Africa - Central)**	**Congo DR**	1	573	520	18%	34%
**AF-NE (Africa - Northeast)**	**Sudan**	3	203	76	88%	100%
	**Uganda**	1	15	12	0%	8%
	**Ethiopia**	2	34	21	0%	0%
	**Kenya, Kisumu**	1	64	63	0%	5%
**AF-E (Africa - East)**	**Kenya, Kilifi**	1	662	627	0%	92%
	**Malawi**	2	371	265	0%	7%
	**Tanzania**	5	697	589	0%	46%
	**Mozambique**	1	91	34	0%	100%
	**Madagascar**	2	25	24	0%	0%
**AS-S-E (Asia - South - East)**	**India, Odisha or West** **Bengal**	2	244	233	100%	100%
**AS-S-FE (Asia - South - Far East)**	**India, Tripura**	1	72	67	100%	100%
	**Bangladesh**	1	1,658	1,310	59%	94%
**AS-SE-W (Asia - Southeast** **- West)**	**Myanmar**	8	1,260	985	69%	79%
	**Thailand, Tak or Ranong**	2	994	895	0%	3%
**AS-SE-E (Asia - Southeast** **- East)**	**Thailand, Sisakhet**	1	112	59	39%	66%
	**Laos**	5	1,052	991	87%	88%
	**Cambodia**	7	1,723	1,267	28%	30%
	**Vietnam**	10	1,733	1,404	62%	84%
**OC-NG (Oceania - New Guinea)**	**Indonesia**	1	133	121	25%	34%
	**Papua New Guinea**	3	251	221	46%	46%
**Total natural infection with** **validated metadata**	**Various locations**	***97***	***20,704***	***16,203***	***42%***	***63%***
**Unverified identity**	**Various locations**	***0***	***160***	***0***		
**Total**		***97***	***20,864***	***16,203***	***42%***	***63%***

The most significant technical advance in the Pf7 dataset is that most of the new samples (12,891/13,752, 94%) came from the MalariaGEN SpotMalaria Project ^7^. SpotMalaria was designed to simplify and standardise the process of collecting dried blood spot (DBS) samples for parasite genetic analysis. The SpotMalaria protocol ensures that the vast majority of DBS samples are suitable for targeted genotyping of drug resistance loci, e.g. by amplicon sequencing, and that a significant proportion of samples are also suitable for whole genome sequencing. This requires an intermediate step known as selective whole genome amplification (sWGA), which makes it possible to obtain parasite genome sequence data from a very small sample, but at the cost of introducing considerable variability in sequencing coverage across the genome. This is the first study to analyse a large number of sWGA *P. falciparum* genomes and therefore it was important to establish that sWGA was not introducing significant biases, and to adapt our methods for calling structural variations which are particularly sensitive to artefactual variation in sequencing coverage.

We have performed a number of analyses to make the Pf7 dataset as useful as possible for a broad range of users. These include descriptions of global population structure, geographic patterns of drug resistance, haplotypic analysis of drug resistance loci, *hrp2* and *hrp3* deletions that can cause failure of rapid diagnostic tests, and variation in the C-terminal of the *csp* antigen used in the most advanced malaria vaccines. These analyses are not intended to be comprehensive and technical users of the dataset can download the analysis-ready dataset for more specialised or detailed investigations.

A high level view of the Pf7 dataset can be obtained from the data exploration tool at the MalariaGEN website. This shows the locations and years where the samples were collected, and the genotype-inferred drug resistance status of each sample. Most importantly it names the investigators who led the studies that contributed the samples at each location and thus made this global dataset possible.

## Results

### Variant discovery and genotyping

We used the Illumina platform to produce whole genome sequencing data on all samples and mapped sequence reads against the *P. falciparum* 3D7 v3 reference genome. The median depth of coverage was 107 sequence reads averaged across the whole genome and across all samples. We used an analysis pipeline for variant discovery and genotyping analogous to that used in Pf6, as outlined in the Methods section.

In the first stage of analysis we discovered genomic variations in nearly half of the 23Mb *P. falciparum* positions (10,145,661 in total, Supplementary Table 3), including 4,397,801 single nucleotide polymorphisms (SNPs).

For the analysis reported here, we excluded all variants in subtelomeric and internal hypervariable regions, mitochondrial and apicoplast genomes and applied stringent quality filters to the remaining variants as described in the Methods section. A total of 3,125,721 SNPs (of which 2,513,888 were biallelic) and 2,742,938 non-SNPs, i.e. short indels or SNP-indel combinations, passed all these filters. Some of the variant positions that were classified as SNPs in Pf6 are now classified as non-SNPs because they additionally include indel alleles.

We performed quality control checks to remove samples with: (i) unverified or incomplete sample collection information; (ii) evidence of co-infection with other *Plasmodium* species; (iii) more than one technical replicate or time course sampling; (iv) low coverage; (v) a higher than expected number of singleton SNPs. In total, we retained 16,203 high-quality samples ( Table 1).

This analysis-ready dataset with details of all participating partner studies and a python package providing convenience methods for accessing is available here.

### Effects of selective whole genome amplification (sWGA)

Unlike the previous version, nearly all samples that are new to this release (12,891/13,752, 94%) have been sequenced after undergoing selective whole genome amplification (sWGA). This process allows us to sequence samples collected as dried blood spots, which greatly simplifies many of the operational challenges in collecting venous blood ^8^.

An artefact introduced by sWGA is high variability in coverage across the genome ^8^. This impacts on the use of local variation in genomic coverage as a way to identify large structural variations such as tandem duplications. We therefore developed a novel method based on GATK GermlineCNVCaller (gCNV) for typing duplications around *mdr1* and *plasmepsin 2–3* (associated with resistance to mefloquine and piperaquine, respectively) and deletions of *hrp2* and *hrp3* (associated with rapid diagnostic test failures). We started by compiling a list of observed breakpoints in and around the loci of interest. We then leveraged on the fact that the amplification bias introduced by sWGA, and the consequent variation in coverage, is relatively systematic and can be used for a cross-sample normalisation. Finally, we complemented the results with an analysis to detect presence of face-away reads around the known breakpoints and obtained a final set of calls. For *plasmepsin 2–3* duplications, concordance between gCNV and the face-away reads methods was high, with 99% of samples called as duplication by gCNV also being called as duplication by the face-away method, and the remaining 1% all called as missing. For *mdr1*, concordance was significantly lower, with 19% of samples called as duplication by gCNV being called as no duplication by the face-away method. This could be explained by the fact that the set of breakpoints used is likely not exhaustive, and also by some duplications not being tandem duplications ^5^. For samples called as no duplication by gCNV, the vast majority were also called as no duplication by the face-away method (83%) or else missing (17%). For *hrp2* and *hrp3* deletions, we manually validated the results and identified evidence of breakpoints for all the deletion calls.

To ensure that sWGA is not introducing biases in population structure, we analysed four sets of samples from the same location and time periods for which we had a substantial number of both sWGA and non-sWGA samples, and could detect no apparent stratification (Supplementary Figure 3).

### Global population structure

**Figure 1.**
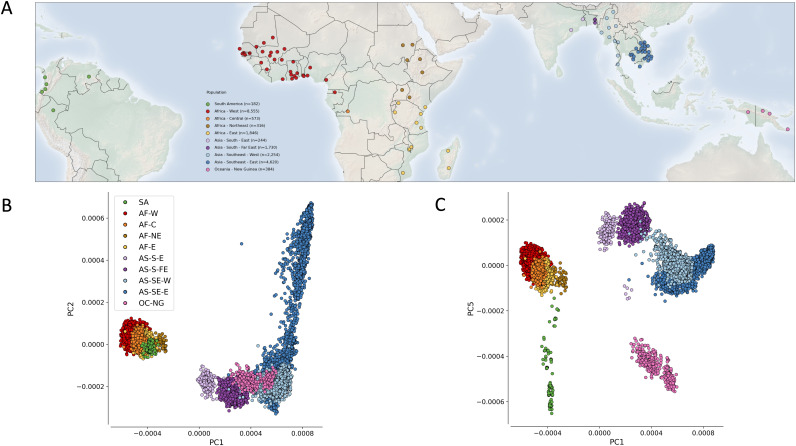
Geographic distribution of sampling locations and population structure. ( **A**) Map shows the centres of the 97 first-level administrative divisions from where samples were collected. Points are coloured according to the major sub-population to which the location is assigned ( Table 1). ( **B**) First two components of a genome-wide principal coordinate analysis. The first axis (PC1, 17.6% of variance explained) captures the separation of African and South American from Asian and Oceanian samples. The second axis (PC2, 2.4% of variance explained) captures finer levels of population structure particularly in the eastern SE Asia population. Each point represents a QC pass sample and the colour legend is the same as in ( **A**). ( **C**) First and fifth (0.7% of variance explained) components of a genome-wide principal coordinate analysis. Here there is an approximate mapping between the principal components and the geographic location (latitude and longitude).

We grouped samples by location using the classification scheme known as first-level administrative division: we refer to these as *sampling locations*. Based on principal coordinate and neighbour-joining tree analyses of all samples, we identified ten major divisions of population structure: we refer to these as *major sub-populations*. We then determined the geographical range of each major sub-population by examining the sampling locations that it contained ( Figure 1 and Supplementary Figure 4). We identified four major sub-populations in Africa: AF-W (8,610 samples from western Africa), AF-C (573 samples from Kinshasa, DRC), AF-NE (316 samples from Sudan, Ethiopia, Uganda, and Kisumu county in western Kenya), AF-E (1,846 samples from east Africa). In Asia we identified four major sub-populations: two in South Asia (AS-S-E, 244 samples from the Indian states of Odisha and West Bengal, and AS-S-FE, 1,730 samples from Bangladesh and the far-eastern Indian state of Tripura), and two in Southeast Asia (AS-SE-W, 2,254 samples from the part of Southeast Asia west of Bangkok, and AS-SE-E, 4,620 samples from the eastern part). The two remaining major sub-populations were OC-NG (384 samples from Oceanian island of New Guinea) and SA (182 samples from South America).

This geographical assignment of ten major sub-populations is a somewhat crude approximation of the underlying population structure, and it does not reflect international conventions for grouping countries or regions. However it provides a framework that allows a broad comparison of population genetic features between different parts of the world, such as the rate of decay of linkage disequilibrium (Supplementary Figure 5), nucleotide diversity (Supplementary Figure 6, lower panel) and complexity of infections (Supplementary Figure 6, upper panel). We also examined the fixation index between sub-populations (Supplementary Figure 7).

### Geographic patterns of drug resistance

We classified samples as resistant or sensitive to major antimalarials and combinations based on genotyping of known drug resistance alleles ( Table 2 - see here for details of the heuristics used). At a regional level, the frequency of samples classified as resistant to each drug is broadly consistent with known and previously reported geographical patterns, with the highest prevalence of multidrug resistance observed in Southeast Asia. Interestingly, in South Asia, we find that the frequency of resistance to chloroquine, sulfadoxine and pyrimethamine appears to be much higher in the far-eastern sub-population (Bangladesh and Tripura) than in the eastern sub-population (Odisha and West Bengal).

**Table 2.** Frequency of different sets of polymorphisms associated with drug resistance in samples from different geographical regions. All samples were classified into different types of drug resistance based on published genetic markers, and represent best attempt based on the available data. Each type of resistance was considered to be either present, absent or unknown for a given sample. For each resistance type, the table reports: the genetic markers considered; the drug they are associated with; the proportion of samples in each major sub-population classified as resistant out of the samples where the type was not unknown. The number of samples classified as either resistant or not resistant varies for each type of resistance considered (e.g. due to different levels of genomic accessibility); numbers in brackets report the minimum and maximum number analysed while the exact numbers considered are reported in Supplementary Table 4. SP: sulfadoxine-pyrimethamine; treatment: SP used for the clinical treatment of uncomplicated malaria; IPTp: SP used for intermittent preventive treatment in pregnancy; AS-MQ: artesunate + mefloquine combination therapy; DHA-PPQ: dihydroartemisinin + piperaquine combination therapy. *dhfr* triple mutant refers to having all three of 51I, 59R and 108N in *dhfr*. *dhfr* and *dhps* sextuple mutant refers to having all five of 51I, 59R and 108N in *dhfr* and 437G and 540E in *dhps*, plus one of *dhfr*:164L, *dhps*:581G, *dhps*:613S or *dhps*:613T. Full details of the rules used to infer resistance status from genetic markers can be found on the resource page at https://www.malariagen.net/resource/34.

Marker	Associated with resistance to	South America (n=154-158)	Africa - West (n=5234-6233)	Africa - Central (n=397-520)	Africa - East (n=1373-1532)	Africa - Northeast (n=120-170)	Asia - South - East (n=164-233)	Asia - South - Far East (n=1212-1369)	Asia - Southeast - West (n=1657-1876)	Asia - Southeast - East (n=2059-3684)	Oceania - New Guinea (n=298-341)
***crt*** **76T**	Chloroquine	100%	29%	61%	24%	40%	31%	94%	99%	95%	96%
***dhfr*** **108N**	Pyrimethamine	64%	87%	100%	96%	98%	64%	100%	100%	99%	99%
***dhps*** **437G**	Sulfadoxine	60%	78%	97%	83%	82%	8%	89%	100%	83%	69%
***mdr1*** **2+ copies**	Mefloquine	0%	0%	0%	0%	0%	0%	0%	29%	5%	1%
***kelch13*** **WHO list**	Artemisinin	0%	0%	0%	0%	0%	0%	0%	36%	58%	1%
***plasmepsin*** ***2-3*** **2+ copies**	Piperaquine	0%	0%	0%	0%	0%	0%	0%	0%	37%	0%
***dhfr*** **triple** **mutant**	SP (treatment)	0%	77%	85%	80%	61%	1%	46%	86%	88%	0%
***dhfr*** **and** ***dhps*** **sextuple** **mutant**	SP (IPTp)	0%	0%	2%	9%	2%	0%	13%	79%	14%	0%
***kelch13*** **and *mdr1***	AS-MQ	0%	0%	0%	0%	0%	0%	0%	10%	4%	0%
***kelch13*** **and** ***plasmepsin*** ***2-3***	DHA-PPQ	0%	0%	0%	0%	0%	0%	0%	0%	35%	0%

**Figure 2.**
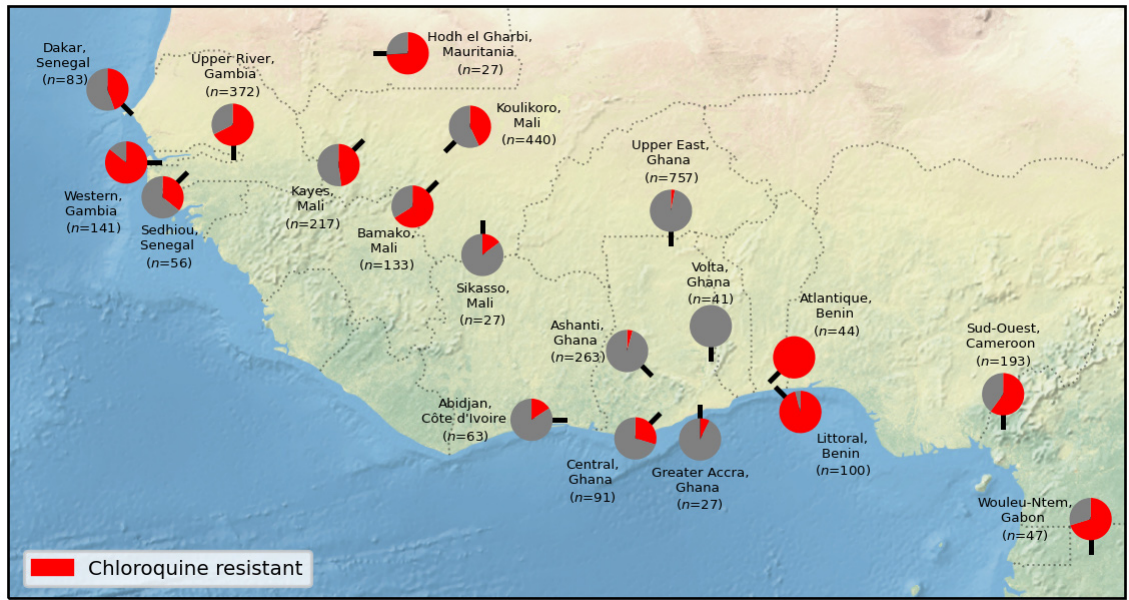
Heterogenity of chloroquine resistance in west Africa. Inferred resistance levels to chloroquine between 2013 and 2016 in different administrative divisions within West Africa. We only include locations for which we have at least 25 samples with an unambiguous inferred chloroquine resistance phenotype. Note the very different chloroquine resistance profiles in nearby locations, e.g. Volta, Ghana vs Atlantique, Benin.

Care is required when interpreting these findings, as most of the major sub-populations spanned a large geographic region, within which there could be considerable epidemiological diversity, and also because we aggregate samples that were collected over relatively long periods of time during which patterns of resistance may have changed (Supplementary Figure 8). To take West Africa as an example, if we consider samples collected between 2013 and 2016 ( Figure 2), we observed levels of chloroquine resistance varying from 0% in Volta, Ghana to 100% in Atlantique, Benin.

Amplifications of the genes *mdr1* and *plasmepsin 2-3* are markers of resistance to mefloquine and piperaquine, respectively. Interestingly, two samples collected in 1993 in Cambodia have tandem duplications of both genes, an event which is relatively rare in more recent samples (only 21 samples in total out of 1,959 that have evidence of amplification of either gene). In addition to presence/absence of the amplification, we also provide details of the associated breakpoints for all samples which shows these two samples having two distinct breakpoints in *plasmepsin*, one of which is identical to that most commonly found in contemporary samples.

### Haplotype analysis of *kelch13* and *crt* drug resistance loci

**Figure 3.**
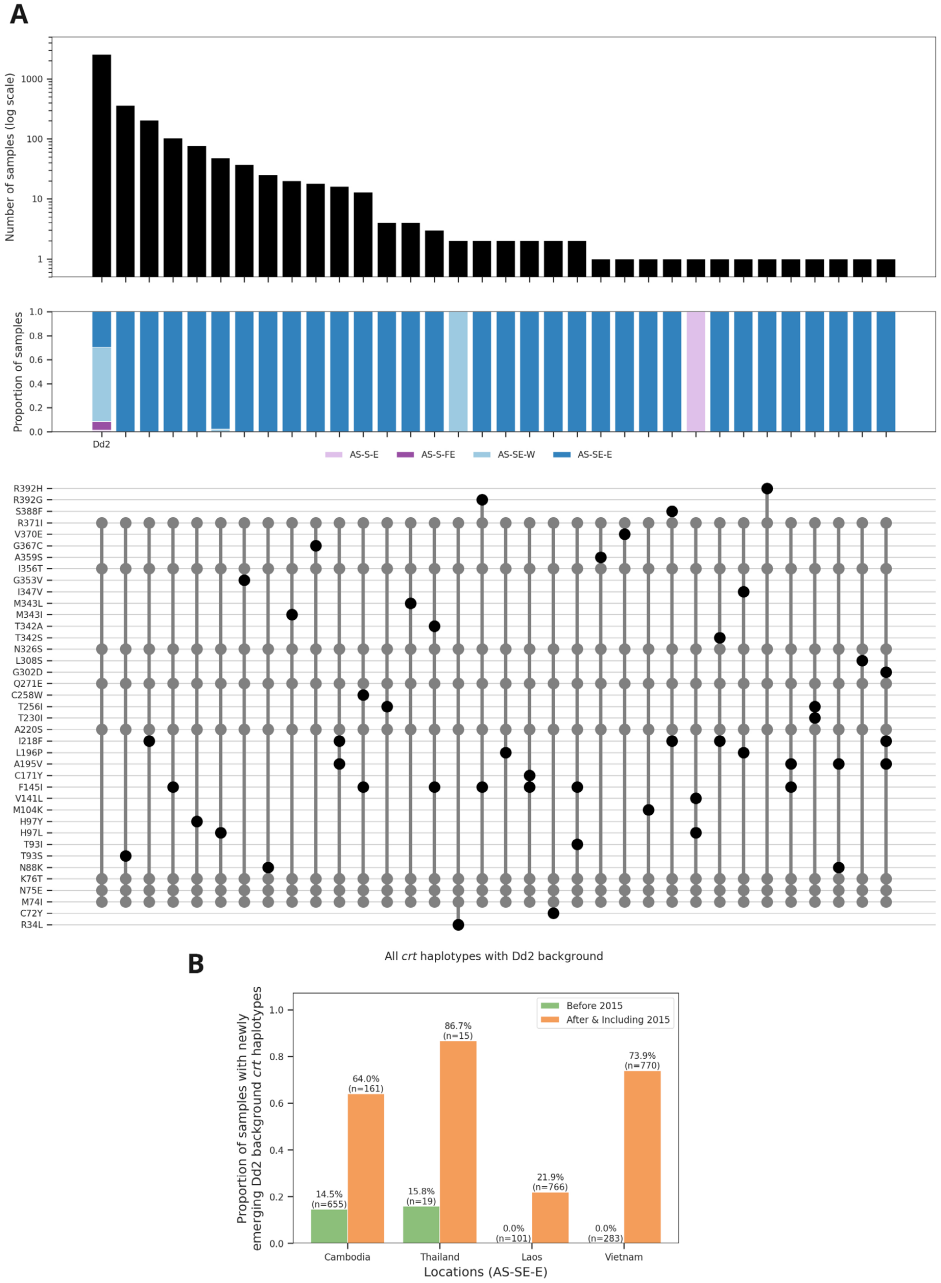
Newly emerging Dd2 background mutations in *crt*. **(a)** Top panel - frequency of different haplotypes with a genetic background identical to the lab strain Dd2. Dd2 is derived from an isolate taken from a patient in Indochina in 1980. Middle panel - breakdown of samples by major sub-population for each haplotype. Lower panel - amino acid mutations in the haplotypes (with respect to 3D7 reference). Mutations found in the Dd2 haplotype are shown in grey, all other mutations are shown in black. **(b)** Bar plots showing changing frequency of newly emerging Dd2 background *crt* haplotypes in different locations in the eastern SE region. Newly emerging Dd2 background haplotypes are defined as all haplotypes that have all mutations seen in Dd2 plus additional mutations.

Previous reports have shown that the current wave of multidrug-resistant *P. falciparum* in Southeast Asia is driven by the KEL1 lineage of the *kelch13* artemisinin resistance locus ^9– 12^ and is associated with multiple new mutations in the *crt* resistance locus ^9^. This dataset confirms the dramatic increase of KEL1 in Cambodia, Eastern Thailand, Laos and Vietnam that has occurred over the past ten years (Supplementary Figure 9). Analysis of the *crt* locus in samples with the K76T resistance variant reveals a major cluster of haplotypes on a common genetic background, the one observed in a widely used lab strain isolated in Asia in 1980 and commonly referred to as Dd2 ( Figure 3A, Supplementary Table 5). In addition to the original Dd2 haplotype, we observe 31 additional mutations. These are essentially restricted to eastern SE Asia with only four samples from outside this region, and they include mutations that have previously been associated with piperaquine resistance ^9, 13^. They are seen across all countries of eastern SE Asia and have risen rapidly in frequency leading us to consider them *newly emerging Dd2-background* haplotypes ( Figure 3B). Most have a single mutation on a Dd2 background, but we observe 13 haplotypes with two mutations and one haplotype (in a single sample) with three mutations (Supplementary Table 5). These findings highlight the value in retrospective analysis of drug resistance mutations, as most of these samples were collected and sequenced before the relevance of *crt* mutations to piperaquine resistance was appreciated.

### Variation in the c-terminal region of *csp*

In addition to the selective pressures due to antimalarial drugs highlighted in the previous section, another area of interest is selective pressure due to vaccines. Having a baseline understanding of genetic variation in vaccine genes is likely to be valuable.

The WHO has recently recommended the RTS,S vaccine for use in regions with moderate to high transmission which includes much of sub-Saharan Africa ^14^. The vaccine targets the gene *csp* and has a construct based on the 3D7 reference sequence of part of the central NANP repeat region where antibodies bind and the c-terminal region which contains T cell epitopes ^15^. Another vaccine based on the same region and sequence, R21, is also showing promise in early stage clinical trials ^16^. Vaccine efficacy is likely to depend on a number of factors, both host and parasite, and clinical trials show some variability between different locations in Africa ^17^. How similar the parasite is in the targeted region to the 3D7 sequence used in vaccine design could be a contributing factor to this variability. Genetic diversity in the construct region may or may not affect vaccine efficacy, and in order to understand this it will be important to monitor efficacy against diversity going forwards. Here we begin a systematic catalogue of population-level diversity in *csp*. While it is challenging if not impossible to genotype the central repeat region using short read data, we start here by looking at variation leading to amino acid changes in the c-terminal region of the protein.

We identified all non-synonymous mutations in the *csp* c-terminal region and analysed the frequency of these in different populations. Interestingly, the vast majority of the samples across the globe carry non-reference alleles, i.e. different from the 3D7 sequence used in the vaccine design, at amino acids 301, 317, 318, 321 and 361 (Supplementary Figure 10). We found a total of 248 unique amino acid haplotypes of *csp*_277-397_ out of 11,254 samples with no ambiguous calls. Amino acid haplotype sequences for the c-terminal region of *csp* for all samples can be found at the MalariaGEN website.

**Figure 4.**
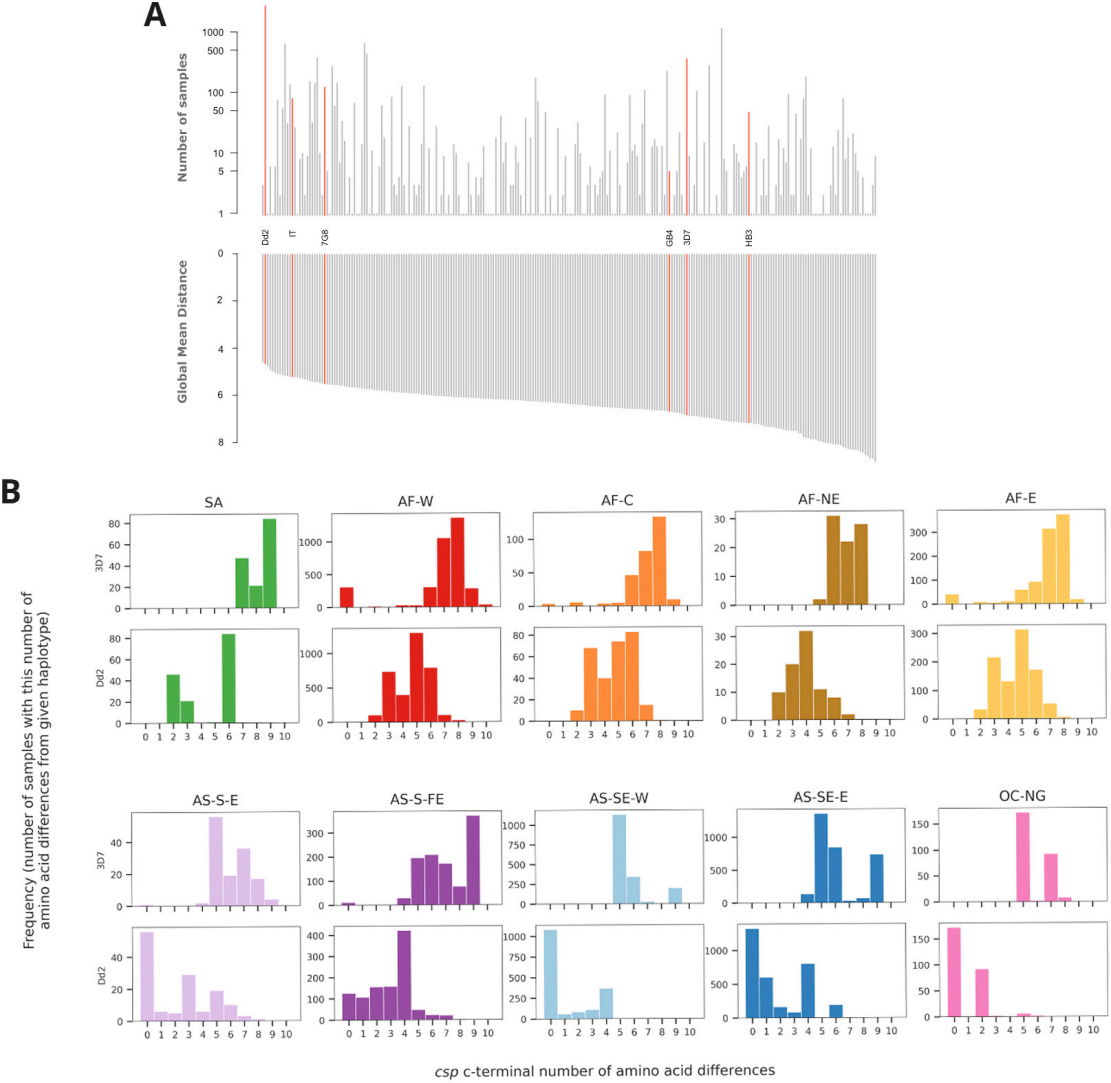
Analysis of c-terminal of *csp*. **(a)** Upper panel - frequency of different haplotypes of c-terminal of *csp*. Haplotypes found in some lab strains are named and highlighted in red. Haplotypes are ordered as per lower panel. Lower panel - global mean distance (number of amino acid differences) to all other haplotypes. **(b)** Histograms of number of amino acid differences between samples in each major sub-population and the 3D7 haplotype (upper plot) and Dd2 haplotype (lower plot).

Surprisingly, the most common haplotype in the dataset and the one with the second lowest number of differences from all other unique haplotypes is the one observed in lab strain Dd2, being found in 2,760/11,254 (25%) of the samples and having a mean of 4.7 differences from other haplotypes ( Figure 4a). In contrast, the 3D7 haplotype used for both *csp*-based vaccines is only found in 3% of samples and has on average 6.9 differences from all other haplotypes.

Importantly, this striking difference also holds when examining each population separately, including in West Africa from where 3D7 is thought to have originated ( Figure 4b).

Taken together, these results show perhaps surprising differences between the target haplotype used in the design of RTS,S and R21 and those circulating in natural parasite populations, and provide a systematic catalogue that can be used in future studies to elucidate any possible clinical significance of sequence diversity.

### Genetic origins of *hrp2* and *hrp3* deletions

Most widely used rapid diagnostic tests (RDTs) rely on detection of the products of the *hrp2* and *hrp3* genes, and deletions in these genes is known to lead to RDT failure ^18^. We used gCNV to call presence/absence of *hrp2* and *hrp3* deletions in 68% of QC passed samples. Frequencies of deletions vary greatly across countries, and deletions of *hrp3* (1.9%) are more common than those of hrp2 (0.14%) (Supplementary Table 6). The only countries where we see deletions in both *hrp2* and *hrp3* that would cause HRP RDTs to fail are Peru (6/20 samples), Indonesia (2/115 samples) and Sudan (1/7 samples).

**Figure 5.**
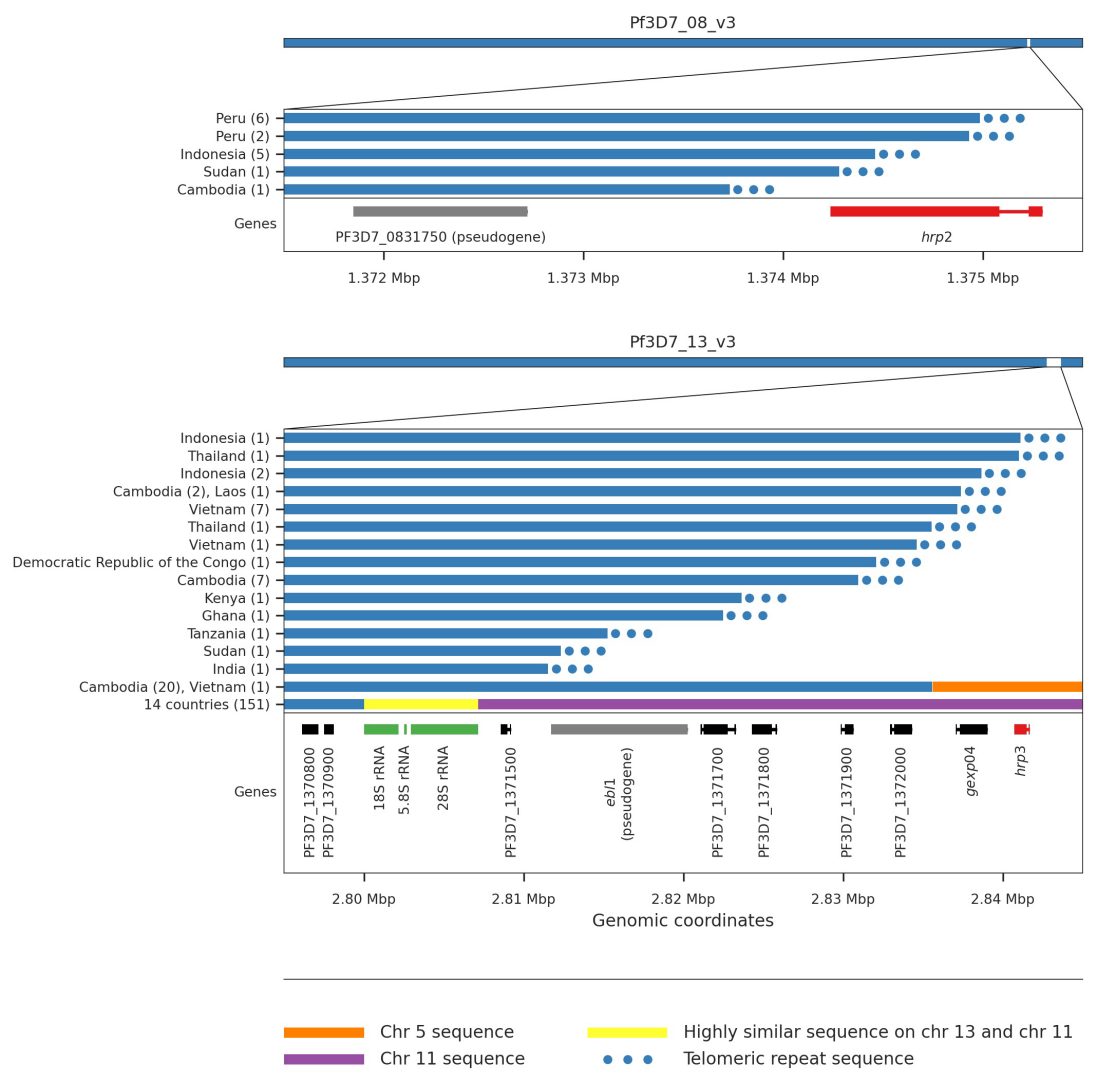
HRP deletion breakpoints. We see five different breakpoints resulting in the deletion of *hrp2*. Four of these are within exon 2 of the gene whereas the fifth is found between *hrp2* and the pseudogene PF3D7_0831750. For all five events we see evidence of telomeric healing from reads that contain part Pf3D7_08_v3 sequence and part telomeric repeat sequence (GGGTTCA/GGGTTTA). We see 16 different breakpoints resulting in the deletion of *hrp3*. For fourteen of these we see evidence of telomeric healing. Note that many of these events result in the deletion of other genes in addition to *hrp3*. For twenty samples from Cambodia and a single sample from Vietnam we see evidence of a recombination with chromosome 5 which results in a hybrid chromosome comprising mostly chromosome 13 sequence but a small inverted section of an internal portion of chromosome 5 containing the gene *mdr1*. We also see evidence of a recombination with chromosome 11 which results in a hybrid chromosome comprising mostly chromosome 13 sequence but also a section of the 3’ end of chromosome 11. This is the most common deletion type, being seen in 151 samples from 14 different countries. Because the recombination occurs between highly similar sequences of a set of three orthologous ribosomal RNA genes found on both chromosomes, it is not possible to identify the exact breakpoints.

There have been numerous reports of such deletions in recent years, but to date there has been little detail on the mechanisms causing such deletions. We manually inspected reads around the apparent breakpoints in order to classify the types of events driving these deletions. For *hrp2*, all deletion events can be explained by a process of telomere healing whereby the end of a chromosome is deleted and a telomere repeat sequence attached to the breakpoint ^19, 20^ ( Figure 5). Telomere healing events can be determined with breakpoint precision and in almost all cases samples with the same breakpoints are from the same country (Supplementary Table 7). For *hrp3*, we also identified a number of telomere healing events, but also two other quite different types of event causing deletion of the gene ( Figure 5, Supplementary Table 7). In many cases a new hybrid chromosome appears to have been created by a recombination between chromosome 13 and 11 at a cluster of rRNA genes that have orthologous copies on both chromosomes. In other cases a recombination between chromosome 13 and an inverted section from within chromosome 5 containing the gene *mdr1* can be identified. This remarkable event results in both deletion of *hrp3* and duplication of *mdr1*.

### Other types of genetic variation: allelic forms of *eba175*

As with previous releases, in Pf7 we have created genotypes at SNPs genome-wide and CNVs in specific locations, but we intend to continue to expand the resource to consider other types of genetic variation. Various surface antigens, including vaccine candidate genes, have two distinctly different allelic forms ^21^. Often the two forms are so divergent that reads from a non-reference form will not map to the 3D7 reference genome ^22^, hence necessitating an alternative to the mapping-based genotype calling approach described above. As an example, the gene *eba175* has two different allelic forms, known as the F- and C-types ^23, 24^. As a proof of concept for such a dimorphic gene, we used a novel kmer-based method to call these two types in each sample. We see that 7,380 (69%) samples have the F allele exclusively and 3,364 (31%) have the C allele. Although frequencies of each type vary between populations, we see >10% frequency of each type in all populations (Supplementary Figure 11). These results give weight to the argument that *eba175* is under balancing selection, most likely negative frequency-dependent selection driven by interactions with the human immune system. Analyses of other such dimorphic genes will be left to future work, as will more detailed analysis of variation within these different allelic types.

## Discussion

The Pf7 dataset increases the amount of curated open data for population genomic analysis of *P. falciparum* by almost threefold, and greatly increases the number of samples collected within the last five years. With denser geographical coverage it is possible to undertake higher resolution analysis of epidemiological variation within a region, e.g. we observe considerable heterogeneity of inferred chloroquine resistance in West Africa ( Figure 3), and it also allows us to identify new sub-populations with distinctive epidemiological features, e.g. we find two sub-populations in south Asia that have contrasting drug resistance profiles. There is useful historical information to be obtained from older samples that are included in this new data release, e.g. some samples collected in Cambodia in the early 1990s appear to be resistant to both piperaquine and mefloquine, which is highly relevant to the ongoing evolution of multidrug resistance to artemisinin combination therapy in Southeast Asia.

An important technical advance is that Pf7 contains a large number of samples that were collected as dried blood spots in the field. We and others have previously described successful whole genome sequencing of *P. falciparum* from DBS after selective whole-genome amplification but it was unclear how well this methodology would perform at scale. Here we show that the SpotMalaria protocol for sWGA of DBS samples allows us to generate whole genome sequence data of sufficient quality to genotype the vast majority of SNPs with sufficient accuracy and reliability for large-scale population genomic analysis. We have introduced improvements to our pipelines for calling copy number variants, necessitated by the greatly increased heterogeneity of sequencing coverage following sWGA. There remain hypervariable gene families and other regions of the parasite genome that cannot be accurately genotyped in field samples using current methods, and these difficulties are compounded by sWGA, but by working on sequencing and analysis methods we aim to continually improve genome coverage in future releases.

The knowledge that DBS samples can be used for whole genome analysis in large-scale studies is of practical importance, as it empowers field researchers and national malaria control programs to integrate population genomic information with other forms of epidemiological and public health data, and it paves the way to a global infrastructure for genomic surveillance of *P. falciparum*. Information about the processes and methods of the SpotMalaria Project can be obtained at the MalariaGEN website

The Pf7 dataset includes a range of analyses and sample annotations that are intended to increase the utility of the data for researchers working on practical problems in malaria control. Compared to the Pf6 dataset, we have made improvements to methods for calling CNVs at the *mdr1* and *pm2* drug resistance loci and for calling *hrp2* and *hrp3* deletions that can affect rapid diagnostic tests. Other new analyses included in Pf7 include more detailed descriptions of: (a) *hrp2* and *hrp3* deletion breakpoints; (b) drug resistance locus haplotypes and in particular newly emerging *crt* haplotypes; (c) profiles of variation in the *csp* vaccine antigen and the vaccine candidate *eba175*. In future releases we aim to improve and expand analyses that are relevant to malaria control programmes and policymakers.

The Pf7 dataset focuses entirely on genome sequencing data, but there is a growing body of data from amplicon sequencing and targeted genotyping approaches that is highly informative about multiple aspects of *P. falciparum* population genomics. For example, the GenRe-Mekong Project has used the SpotMalaria platform combined with amplicon sequencing to enable malaria control programmes in the Greater Mekong Region to conduct national genomic surveillance of multidrug resistance ^6^. In future data releases we aim to integrate data from these different sources to greatly increase sample size and geographical coverage, and thus improve the resolution of population genomic analysis.

## Methods

All samples in this study were derived from blood samples obtained from patients with *P. falciparum* malaria, collected with informed consent from the patient or a parent or guardian. At each location, sample collection was approved by the appropriate local and institutional ethics committees. The following local and institutional committees gave ethical approval for the partner studies: Walter and Eliza Hall Institute Human Research Ethics Committee, Australia; University of Antwerp, Belgium; Comite d'Ethique de la Recherche - Institut des Sciences Biomedicales Appliquees, Benin; Ministere de la Sante – Republique du Benin, Benin; Comité d'Éthique, Ministère de la Santé, Bobo-Dioulasso, Burkina Faso; Ministry of Health National Ethics Committee for Health Research, Cambodia; Institutional Review Board University of Buea, Cameroon; Comite Institucional de Etica de investigaciones en humanos de CIDEIM, Colombia; Research Ethics Committee of the Faculty of Medicine of the National University of Colombia; Comité National d'Ethique de la Recherche, Cote d’Ivoire; Comite d’Ethique Universite de Kinshasa, Democratic Republic of Congo; Armauer Hansen Research Institute Institutional Review Board, Ethiopia; Addis Ababa University, Aklilu Lemma Institute of Pathobiology Institutional Review Board, Ethiopia; Ghana Health Service Ethical Review Committee, Ghana; University of Ghana Noguchi Medical Research Institute, Ghana; Navrongo Health Research Centre Institutional Review Board, Ghana; Comite d’Ethique National Pour la Recherché en Santé, République de Guinée; KEMRI Scientific and Ethics Review Unit, Kenya; Ministry of Health National Ethics Committee For Health Research, Laos; College of Medicine, University of Lagos, Nigeria; Comité National d'Ethique auprès du Ministère de la Santé Publique, Madagascar; College of Medicine Regional Ethics Committee University of Malawi, Malawi; Faculté de Médecine, de Pharmacie et d'Odonto-Stomatologie, University of Bamako, Bamako, Mali; L'université des Sciences, des Techniques et des Technologies de Bamako, Mali; Ethics Committee of the Ministry of Health, Mali; Ethics committee of the Ministry of Health, Mauritania; National Bioethics Committees of Mozambique (CNBS); Institutional Review Board, Papua New Guinea Institute of Medical Research, Goroka, Papua New Guinea; PNG Medical Research Advisory Council (MRAC), Papua New Guinea; Institutional Review Board, Universidad Nacional de la Amazonia Peruana, Iquitos, Peru; Al Neelain University Institutional Review Board, Sudan; Federal Ministry of Health, Sudan; Ethics Committee of the Ministry of Health, Senegal; Medical Research Coordinating Committee of the National Institute for Medical Research, Tanzania; Ethics Committee, Faculty of Tropical Medicine, Mahidol University, Bangkok, Thailand; Gambia Government/MRC Joint Ethics Committee, Banjul, The Gambia; Liverpool School of Tropical Medicine, UK; London School of Hygiene and Tropical Medicine Ethics Committee, London, UK; Oxford Tropical Research Ethics Committee, Oxford, UK; University College London Hospitals Research Ethics Committee, UK; Walter Reed Army Institute of Research, USA; National Institute of Allergy and Infectious Diseases, Bethesda, MD, USA; University of Maryland School of Medicine IRB, USA; Ministry of Health Institute of Malariology-Parasitology-Entomology, Vietnam.

Standard laboratory protocols were used to determine DNA quantity and proportion of human DNA in each sample as previously described ^22, 25^.

Here we summarise the bioinformatics methods used to produce and analyse the data; full details are available here.

Reads mapping to the human reference genome were discarded before all analyses, and the remaining reads were mapped to the *P. falciparum* 3D7 v3 reference genome using bwa mem ^26^. “Improved” BAMs were created using the Samtools FixMateInformation, Picard MarkDuplicates, and GATK base quality score recalibration. All lanes for each sample were merged to create sample-level BAM files.

Putative variants were called in each sample independently using GATK (v4.1.4.0) HaplotypeCaller, then all samples were combined to jointly genotype the entire cohort using GATK GenotypeGVCFs ^27^.

SNPs and indels were filtered using GATK’s Variant Quality Score Recalibration (VQSR). Variants with a VQSLOD score ≤ 2 were filtered out. Functional annotations were applied using snpEff ^28^ version 4.3. Genome regions were annotated using BCFtools v1.10.2 ( http://www.htslib.org/doc/bcftools.html) and masked if they were outside the core genome. Unless otherwise specified, we used biallelic SNPs that pass all quality filters for all the analysis.

VCF files were converted to zarr v2.4.0 format and subsequent analyses were mainly performed using scikit-allel v1.2.1 and the zarr files.

We identified species using nucleotide sequence from reads mapping to six different loci in the mitochondrial genome, using custom java code. The loci were located within the *cox3* gene (PF3D7_MIT01400), as described in a previously published species detection method ^29^. Alleles at various mitochondrial positions within the six loci were genotyped and used for classification.

We created a final analysis set of 16,203 samples after removing samples with unverified identity, mixed species, replicate and low coverage samples, and samples with excessive numbers of singleton SNPs.

We calculate genetic distance between samples using biallelic coding SNPs that pass filters using a method previously described ^9^.

The matrix of genetic distances was used to generate neighbour-joining trees and principal coordinates. Based on these observations we grouped the samples into ten major sub-populations: South America, West Africa, Central Africa, Northeast Africa, East Africa, an eastern part of South Asia, a far-eastern part of South Asia, the western part of Southeast Asia, the eastern part of Southeast Asia and Oceania, with samples assigned to region based on the geographic location of the sampling site.

*F _WS_* was calculated using custom python scripts using the method previously described ^7^. Nucleotide diversity (π) was calculated in non-overlapping 25kbp genomic windows, only considering coding SNPs to reduce the ascertainment bias caused by poor accessibility of non-coding regions. LD decay ( *r* ^2^) was calculated using the method of Rogers and Huff and biallelic SNPs with low missingness and regional allele frequency >10%. Mean *F _ST_* between populations was calculated using Hudson’s method.

To call duplication genotypes around *mdr1* and *plasmepsin 2-3* from binned coverage, we adapted the GATK GermlineCNVCaller (gCNV) pipeline, which features a probabilistic Bayesian model that jointly infers both copy-number activity and a model for denoising sequencing systematics. After breakpoint genotypes were called, we performed an initial, permissive round of annotation-based call filtering, using hard cuts to identify failing samples and demarcate duplication and reference genotypes. This was followed by a final round of curation, based on manual inspection of the denoised copy ratios, to discard spurious duplication calls. The resulting filtered gCNV call set was integrated with an analogous call set based on consideration of face-away read-pair evidence, in which we set the breakpoint to be that with the highest proportion of face-away reads.

Deletions in *hrp2* and *hrp3*, genes which are located in subtelomeric regions of the genome with very high levels of natural variation, were identified using the same breakpoint-genotyping framework introduced above. As before, an initial round of permissive, annotation-based filtering was performed, followed by a final round of curation to discard spurious deletion calls. We identified deletion breakpoints by manual inspection of custom plots.

The procedure used to map genetic markers to inferred resistance status classification is described in detail for each drug in the accompanying data release. In brief, we called amino acids at selected loci by first determining the reference amino acids and then, for each sample, applying all variations using the GT field of the VCF file. This same approach was used to identify haplotypes of *csp*_277-397_. The amino acid and copy number calls generated in drug resistance genes were used to classify all samples into different types of drug resistance. Our methods of classification were heuristic and based on the available data and current knowledge of the molecular mechanisms. Each type of resistance was considered to be either present, absent or unknown for a given sample. *eba175* F- and C-type calls are made by identifying samples that have 19bp kmers present that are unique to the C and F haplotypes.

## Data Availability

Data are available under the MalariaGEN terms of use for the Pf Community Project: https://www.malariagen.net/data/terms-use/p-falciparum-community-project-terms-use. Depending on the nature, format and content of the data, appropriate mechanisms have been utilised for data access, as detailed below. This project contains the following underlying data that are available as an online resource: https://www.malariagen.net/resource/34. Data are also available from Figshare. Figshare: Supplementary data to: Pf7: an open dataset of Plasmodium falciparum genome variation in 20,000 worldwide samples. https://doi.org/10.6084/m9.figshare.21674321 ^30^. Study information: Details of the 82 contributing partner studies, including description, contact information and key people. Sample provenance and sequencing metadata: sample information including partner study information, location and year of collection, ENA accession numbers, and QC information for 20,864 samples from 33 countries. Measure of complexity of infections: characterisation of within-host diversity ( *F _WS_*) for 16,203 QC pass samples. Drug resistance marker genotypes: genotypes at known markers of drug resistance for 16,203 samples, containing amino acid and copy number genotypes at six loci: crt, dhfr, dhps, mdr1, kelch13, plasmepsin 2-3. Inferred resistance status classification: classification of 16,203 QC pass samples into different types of resistance to 10 drugs or combinations of drugs and to RDT detection: chloroquine, pyrimethamine, sulfadoxine, mefloquine, artemisinin, piperaquine, sulfadoxine- pyrimethamine for treatment of uncomplicated malaria, sulfadoxine- pyrimethamine for intermittent preventive treatment in pregnancy, artesunate-mefloquine, dihydroartemisinin-piperaquine, hrp2 and hrp3 gene deletions. Drug resistance markers to inferred resistance status: details of the heuristics utilised to map genetic markers to resistance status classification. CRT haplotypes: Full *crt* gene haplotypes for 16,203 QC pass samples. CSP C-terminal haplotypes: Full *csp* C-terminal haplotypes for 16,203 QC pass samples plus 6 lab strains. EBA175 calls: *eba175* allelic type calls for 16,203 QC pass samples. Reference genome: the version of the 3D7 reference genome fasta file used for mapping. Annotation file: the version of the 3D7 reference annotation gff file used for genome annotations. Genetic distances: Genetic distance matrix comparing all 20,864 samples. Short variants genotypes: Genotype calls on 10,145,661 SNPs and short indels in all 20,864 samples from 33 countries, available both as VCF and zarr files. Data are available under the terms of the Creative Commons Attribution 4.0 International license (CC-BY 4.0).

## References

[1] World malaria report 2021. Reference Source

[2] Neafsey DE Taylor AR MacInnis BL : Advances and opportunities in malaria population genomics. *Nat Rev Genet.* 2021;22(8):502–517. 10.1038/s41576-021-00349-5 33833443 PMC8028584

[3] Malaria Genomic Epidemiology Network: A global network for investigating the genomic epidemiology of malaria. *Nature.* 2008;456(7223):732–737. 10.1038/nature07632 19079050 PMC3758999

[4] https://www.malariagen.net/parasite/pf3k

[5] MalariaGEN: Ahouidi A Ali M : An open dataset of *Plasmodium falciparum* genome variation in 7,000 worldwide samples [version 2; peer review: 2 approved]. *Wellcome Open Res.* 2021;6:42. 10.12688/wellcomeopenres.16168.2 33824913 PMC8008441

[6] Jacob CG Thuy-Nhien N Mayxay M : Genetic surveillance in the Greater Mekong subregion and South Asia to support malaria control and elimination. *eLife.* 2021;10:e62997. 10.7554/eLife.62997 34372970 PMC8354633

[7] https://www.malariagen.net/parasite/spotmalaria

[8] Oyola SO Ariani CV Hamilton WL : Whole genome sequencing of *Plasmodium falciparum* from dried blood spots using selective whole genome amplification. *Malar J.* 2016;15(1):597. 10.1186/s12936-016-1641-7 27998271 PMC5175302

[9] Hamilton WL Amato R van der Pluijm RW : Evolution and expansion of multidrug-resistant malaria in southeast Asia: a genomic epidemiology study. *Lancet Infect Dis.* 2019;19(9):943–951. 10.1016/S1473-3099(19)30392-5 31345709 PMC6715858

[10] Amato R Pearson RD Almagro-Garcia J : Origins of the current outbreak of multidrug-resistant malaria in southeast Asia: a retrospective genetic study. *Lancet Infect Dis.* 2018;18(3):337–345. 10.1016/S1473-3099(18)30068-9 29398391 PMC5835763

[11] Imwong M Suwannasin K Kunasol C : The spread of artemisinin-resistant *Plasmodium falciparum* in the Greater Mekong subregion: a molecular epidemiology observational study. *Lancet Infect Dis.* 2017;17(5):491–497. 10.1016/S1473-3099(17)30048-8 28161569 PMC5406483

[12] Imwong M Hien TT Thuy-Nhien NT : Spread of a single multidrug resistant malaria parasite lineage ( *PfPailin*) to Vietnam. *Lancet Infect Dis.* 2017;17(10):1022–1023. 10.1016/S1473-3099(17)30524-8 28948924

[13] Wicht KJ Mok S Fidock DA : Molecular Mechanisms of Drug Resistance in *Plasmodium falciparum* Malaria. *Annu Rev Microbiol.* 2020;74:431–454. 10.1146/annurev-micro-020518-115546 32905757 PMC8130186

[14] WHO recommends groundbreaking malaria vaccine for children at risk. Reference Source

[15] Heppner DG Kester KE Ockenhouse CF : Towards an RTS,S-based, multi-stage, multi-antigen vaccine against falciparum malaria: progress at the Walter Reed Army Institute of Research. *Vaccine.* 2005;23(17–18):2243–2250. 10.1016/j.vaccine.2005.01.142 15755604

[16] Datoo MS Natama HM Somé A : High Efficacy of a Low Dose Candidate Malaria Vaccine, R21 in 1 Adjuvant Matrix-M ^TM^, with Seasonal Administration to Children in Burkina Faso.2021. 10.2139/ssrn.3830681 PMC8121760 33964223

[17] RTS,S Clinical Trials Partnership: Efficacy and safety of RTS,S/AS01 malaria vaccine with or without a booster dose in infants and children in Africa: final results of a phase 3, individually randomised, controlled trial. *Lancet.* 2015;386(9988):31–45. 10.1016/S0140-6736(15)60721-8 25913272 PMC5626001

[18] WHO: False-negative RDT results and implications of new reports of *P. falciparum histidine-rich protein 2/3* gene deletions: information note. *False-Negat RDT Results Implic New Rep P Falciparum Histidine-Rich Protein 23 Gene Deletions Inf Note.* 2016. Reference Source

[19] Scherf A Mattei D : Cloning and characterization of chromosome breakpoints of *Plasmodium falciparum*: breakage and new telomere formation occurs frequently and randomly in subtelomeric genes. *Nucleic Acids Res.* 1992;20(7):1491–1496. 10.1093/nar/20.7.1491 1579440 PMC312228

[20] Zhang X Alexander N Leonardi I : Rapid antigen diversification through mitotic recombination in the human malaria parasite *Plasmodium falciparum*. *PLoS Biol.* 2019;17(5):e3000271. 10.1371/journal.pbio.3000271 31083650 PMC6532940

[21] Roy SW Ferreira MU Hartl DL : Evolution of allelic dimorphism in malarial surface antigens. *Heredity (Edinb).* 2008;100(2):103–110. 10.1038/sj.hdy.6800887 17021615

[22] Miles A Iqbal Z Vauterin P : Indels, structural variation, and recombination drive genomic diversity in *Plasmodium falciparum*. *Genome Res.* 2016;26(9):1288–1299. 10.1101/gr.203711.115 27531718 PMC5052046

[23] Ware LA Kain KC Lee Sim BK : Two alleles of the 175-kilodalton *Plasmodium falciparum* erythrocyte binding antigen. *Mol Biochem Parasitol.* 1993;60(1):105–109. 10.1016/0166-6851(93)90033-t 8366884

[24] Wendler JP : Accessing complex genomic variation in Plasmodium falciparum natural infections. (Oxford University, UK, 2015). Reference Source

[25] Manske M Miotto O Campino S : Analysis of *Plasmodium falciparum* diversity in natural infections by deep sequencing. *Nature.* 2012;487(7407):375–379. 10.1038/nature11174 22722859 PMC3738909

[26] Li H Durbin R : Fast and accurate short read alignment with Burrows–Wheeler transform. *Bioinformatics.* 2009;25(14):1754–1760. 10.1093/bioinformatics/btp324 19451168 PMC2705234

[27] DePristo MA Banks E Poplin R : A framework for variation discovery and genotyping using next-generation DNA sequencing data. *Nat Genet.* 2011;43(5):491–498. 10.1038/ng.806 21478889 PMC3083463

[28] Cingolani P Platts A Wang LL : A program for annotating and predicting the effects of single nucleotide polymorphisms, *SnpEff: SNPs in the genome of Drosophila melanogaster strain w ^1118^; iso-2; iso-3*. *Fly (Austin).* 2012;6(2):80–92. 10.4161/fly.19695 22728672 PMC3679285

[29] Echeverry DF Deason NA Davidson J : Human malaria diagnosis using a single-step direct-PCR based on the *Plasmodium* cytochrome oxidase III gene. *Malar J.* 2016;15: 128. 10.1186/s12936-016-1185-x 26928594 PMC4772515

[30] MalariaGEN: Pf7: an open dataset of Plasmodium falciparum genome variation in 20,000 worldwide samples. *figshare* . Dataset.2022. 10.6084/m9.figshare.21674321.v2. PMC9971654 36864926

