# Supplementary material for: Pf7: an open dataset of Plasmodium falciparum genome variation in 20,000 worldwide samples
Source: Wellcome Open Res. 2023 Jan 16;8:22. [Version 1] doi: 10.12688/wellcomeopenres.18681.1 (PMC9971654; doi:10.12688/wellcomeopenres.18681.1)
Supplement: Peer review history

# Peer review: Reviewer response for version 1

**DOI:** 10.21956/wellcomeopenres.20716.r54149

**Published:** 2023-02-21

**Authors:** Cristian Koepfli

**Affiliations:** 1 Department of Biological Sciences, Eck Institute for Global Health, University of Notre Dame, Notre Dame, IN, USA

This manuscript presents the latest version of the MalariaGEN P. falciparum dataset, with over 20,000 whole genomes. Given the very rich dataset, the manuscript necessarily needs to focus on brief analysis of a few key aspects, namely the diversity and population structure of markers of drug resistance, hrp2/3 deletion, and (potential) vaccine candidates csp and eba175.

 While the results are overall clearly described despite the brevity, the global population structure shown in Figure 1 is difficult to follow. The authors define ten major sub-populations. Yet, in both PCoA plots shown, the separation of several populations is not apparent. For example, the four African populations seem to overlap, as do the two populations from SE-Asia. In contrast, the Oceania-New Guinea population seems to split into two (I am surprised there is no comment on that). Given the grouping of the sub-populations is maintained to describe the analysis throughout the manuscript, the authors might consider adding panels with further components to better show separation of sub-populations. Or, maybe, a PCoA with African isolates only might make the separation more evident.

 As further point for clarification, in the section on sWGA the authors the analysis of face-away reads. This term seems not to be commonly known among people working with WGS data. Do the authors mean reads spanning the break-points? This section seems rather technical compared to the remainder of the manuscript.

Is the work clearly and accurately presented and does it cite the current literature?

Yes

If applicable, is the statistical analysis and its interpretation appropriate?

Yes

Are all the source data underlying the results available to ensure full reproducibility?

Yes

Is the study design appropriate and is the work technically sound?

Yes

Are the conclusions drawn adequately supported by the results?

Yes

Are sufficient details of methods and analysis provided to allow replication by others?

Yes

Reviewer Expertise:

Molecular epidemiology, diagnosis of P. falciparum and P. vivax, genotyping, amplicon sequencing, hrp2/2 deletion typing, gametocyte quantification, transmission

I confirm that I have read this submission and believe that I have an appropriate level of expertise to confirm that it is of an acceptable scientific standard.

# Peer review: Reviewer response for version 1

**DOI:** 10.21956/wellcomeopenres.20716.r54145

**Published:** 2023-02-07

**Authors:** David A. Fidock

**Affiliations:** 1 Department of Microbiology and Immunology, Columbia University Irving Medical Center, New York, NY, USA

This study by the MalariaGEN consortium and multiple partners across the malaria-endemic world is exceptional. The authors report the results of a remarkable study that genetically profiled over 20,000 Plasmodium falciparum samples in the current Pf7 database, including an additional 13,752 samples compared to their most recent data iteration (Pf6), adding an additional 33 partner sites and five countries. The study contains whole-genome sequencing data from over 16,000 samples, which required selective whole-genome amplification (sWGA) to generate sufficient data reads from the limited amounts of parasite DNA that could be recovered from dried blood spots. The authors describe a careful optimization and analysis of the methodology employed, as sWGA creates some uneven distribution in the sample reads across the genome. The authors conclude that this method is sufficiently reliable that it enables calling copy number variants across the genome, which is a new and important feature to this new analysis and data release.

 The published article version highlights a few of the most important findings, including:

- Figure 1: population sub-structuring across their samples.
- Figure 2: Heterogeneity of chloroquine-resistant P. falciparum across west Africa (as determined using the pfcrt K76T mutation that is a well validated genetic marker). This finding likely reflects different chloroquine and potentially amodiaquine usage across the region.
- Figure 3: The finding of multiple new pfcrt mutations in Southeast Asia, arising on the chloroquine-resistant Dd2 background, which in a number of cases reflects a key role for these mutations in contributing to piperaquine resistance. A number of these mutations are also likely to compensate for fitness costs caused by pfcrt mutations.
- Figure 4: Evidence of a high rate of non-synonymous mutations in CSP, which is important given that a segment of this protein forms the core parasite antigen used in the WHO-approved RTS,S and the clinically trialed R21 vaccines. That figure would benefit from additional annotation to show the location of the segments used in these vaccines.
- Figure 5: Evidence of breakpoints that cause deletions of HRP2 and HRP3, whose detection is an essential component of the rapid diagnostic tests commonly used to diagnose malarial infection. Here, it would be good to point out the absence of samples from the Horn of Africa, where this is a major problem. That description should cite the relevant literature, e.g. PMID 34580442 ^1^.
- Table 1 lists the new samples by region.
- Table 2 is a very useful list of the prevalence of genetic markers of resistance to multiple drugs. When citing amplification of plasmepsins 2 and 3 as a marker of piperaquine resistance, the authors should cite the two initial reports of their association: PMID 27818095 ^2^ and 27818097 ^3^. This lists, by necessity, excludes pfcrt mutations as, unlike for chloroquine, there is no single mutation that is essential and instead there are several that can contribute to resistance. The authors cite a review but could include a recent study that provides more insight into mechanism: PMID 31776516 ^4^.

The real power of this study can be found through their searchable website: https://www.malariagen.net/resource/34. This resource will provide a superb tool for the malaria research community. Now that this article is published, the following link can be updated to reflect that data release should no longer be forthcoming: https://www.malariagen.net/apps/pf7/. It will be important for readers to be able to interrogate the database in an easy way to collate information on variation in a particular gene. I was not able yet to figure out how to generate this search and more instruction on this point would be very helpful.

 This is a remarkable study in the history of malaria research. The entire team should be commended for this extraordinary dataset.

Is the work clearly and accurately presented and does it cite the current literature?

Yes

If applicable, is the statistical analysis and its interpretation appropriate?

Not applicable

Are all the source data underlying the results available to ensure full reproducibility?

Yes

Is the study design appropriate and is the work technically sound?

Yes

Are the conclusions drawn adequately supported by the results?

Yes

Are sufficient details of methods and analysis provided to allow replication by others?

Yes

Reviewer Expertise:

Malaria, drug resistance, therapeutics mode of action, genetics, mechanism, gene editing, Plasmodium falciparum,

I confirm that I have read this submission and believe that I have an appropriate level of expertise to confirm that it is of an acceptable scientific standard.

## References

[1] : Plasmodium falciparum is evolving to escape malaria rapid diagnostic tests in Ethiopia. *Nat Microbiol* .2021;6(10) : 10.1038/s41564-021-00962-4 1289-1299 10.1038/s41564-021-00962-4 34580442PMC8478644

[2] : Genetic markers associated with dihydroartemisinin-piperaquine failure in Plasmodium falciparum malaria in Cambodia: a genotype-phenotype association study. *Lancet Infect Dis* .2017;17(2) : 10.1016/S1473-3099(16)30409-1 164-173 10.1016/S1473-3099(16)30409-1 27818095PMC5564489

[3] : A surrogate marker of piperaquine-resistant Plasmodium falciparum malaria: a phenotype-genotype association study. *Lancet Infect Dis* .2017;17(2) : 10.1016/S1473-3099(16)30415-7 174-183 10.1016/S1473-3099(16)30415-7 27818097PMC5266792

[4] : Structure and drug resistance of the Plasmodium falciparum transporter PfCRT. *Nature* .2019;576(7786) : 10.1038/s41586-019-1795-x 315-320 10.1038/s41586-019-1795-x 31776516PMC6911266

# Peer review: Reviewer response for version 1

**DOI:** 10.21956/wellcomeopenres.20716.r54147

**Published:** 2023-02-02

**Authors:** Fabián Sáenz

**Affiliations:** 1 Centro de Investigación para la Salud en América Latina, Facultad de Ciencias Exactas y Naturales, Pontificia Universidad Católica del Ecuador, Quito, Ecuador

This manuscript by MalariaGEN and collaborators describes a new release set of genomic data called Pf7. The data includes curated genomic data of more than 20,000 *Plasmodium falciparum* samples, out of which 13750 were new for the Pf7 set. Samples were obtained from 33 countries in 4 continents collected between 1984 and 2018. Most of the new samples came from dried blood spots for which SWGA was used (and used a GATK based method to normalize data around certain genes). Variants were called and incomplete samples discarded. The authors divided the samples in 10 sub populations that matched quite well sample locations based in principal coordinate analysis and neighbor joining trees (four in Africa, four in Asia, one in Oceania and one in South America). In order to show relevance of the data, some interesting analysis specific to drug resistance, vaccine target diversity, rapid diagnosis test target deletion and invasion ligands was presented. Of particular interest, samples were classified according to the resistance level to some of the main drugs using well known markers and show how drug resistance has moved across SE Asia in unique genetic backgrounds. In addition, the variation in the c-terminal region and background mutations was analyzed and shown to be quite divergent from the reference 3D7 (used in the RTS,S vaccine). In addition to map the deletions of *hrp2* and *hrp3* in the samples around the world. the origin of these deletions is explored and the authors conclude that telomere healing plays an important role in the process. The available data also helps confirm that eba175 is an invasion ligand gene under balancing selection.

 This work is a very important addition to the genomic *Plasmodium* datasets available for the scientific community. The data will help the development of new worldwide and regional malaria studies helping elimination in several areas of the world and ultimately helping in the ultimate goal that is eradication.

 Here are some minor observations to this work: 

 Introduction:

- The analysis pipeline should be referenced through the text.

Results:

- Variant discovery and genotyping: When referring to 3D7 v3 reference genome a reference should be included. When referring to the variant discovery pipeline for Pf6, a reference should be included. 

Effects of selective whole genome amplification (sWGA):

- The authors are well aware that variability in coverage across the genome can arise from sWGA and develop a method based on gCNV. It can be seen that for some genes such as mdr1, concordance was low. Would this method be usable for other markers in the genome? Limitations in this sense should be further discussed.
- To show that sWGA of DBS samples is not introducing a bias in population structure, the authors compare clustering of the same samples from whole blood and DBS. They do not see stratification by sample type (supl fig 3). From this figure, it is not clear to me that clustering is similar in both cases since samples of both collection types are analysed together. In addition, it is not clear how sample type clustering would be sufficient to demonstrate no bias in population structure due to sWGA. It would be important to have further insight from the authors in this sense.

Global population structure:

- In figure 1B and supplementary figure 4 it is of particular interest that some African samples and South American isolates appear to be more related than African samples between themselves, but this is not commented by the authors. Could the authors comment in this sense.

Genetic origins of *hrp2* and *hrp3* deletions: 

- The first sentence in this section reads: “Most widely used rapid diagnostic tests rely on detection of the products of the hrp2 and hrp3 genes”. In my opinion this should be clarified or written differently since RDTs are designed to detect the product of *hrp2* gene but no *hrp3*. The deletion of hrp3 may affect the outcome of RDTs if *hrp2* is deleted because of the similarity between both of the products.

Is the work clearly and accurately presented and does it cite the current literature?

Yes

If applicable, is the statistical analysis and its interpretation appropriate?

Yes

Are all the source data underlying the results available to ensure full reproducibility?

Yes

Is the study design appropriate and is the work technically sound?

Yes

Are the conclusions drawn adequately supported by the results?

Yes

Are sufficient details of methods and analysis provided to allow replication by others?

Yes

Reviewer Expertise:

Infectious diseases: Malaria genetics and genomics

I confirm that I have read this submission and believe that I have an appropriate level of expertise to confirm that it is of an acceptable scientific standard.
